# Allele-specific suppression in *Caenorhabditis elegans* reveals details of EMS mutagenesis and a possible moonlighting interaction between the vesicular acetylcholine transporter and ERD2 receptors

**DOI:** 10.1093/genetics/iyab065

**Published:** 2021-04-29

**Authors:** Eleanor A Mathews, Dave Stroud, Gregory P Mullen, Gavriil Gavriilidis, Janet S Duerr, James B Rand, Jonathan Hodgkin

**Affiliations:** 1 Oklahoma Medical Research Foundation, Oklahoma City, OK 73104, USA; 2 Department of Biochemistry, University of Oxford, Oxford OX1 3QU, UK; 3 Department of Biological Sciences, Ohio University, Athens, OH 45701, USA; 4 Oklahoma Center for Neuroscience, Oklahoma City, OK 73104, USA

**Keywords:** Caenorhabditis elegans, synapse, acetylcholine transporter, genetic suppression, KDEL receptor, protein interaction, EMS mutagenesis, moonlighting

## Abstract

A missense mutant, *unc-17(e245)*, which affects the *Caenorhabditis elegans* vesicular acetylcholine transporter UNC-17, has a severe uncoordinated phenotype, allowing efficient selection of dominant suppressors that revert this phenotype to wild-type. Such selections permitted isolation of numerous suppressors after EMS (ethyl methanesulfonate) mutagenesis, leading to demonstration of delays in mutation fixation after initial EMS treatment, as has been shown in T4 bacteriophage but not previously in eukaryotes. Three strong dominant extragenic suppressor loci have been defined, all of which act specifically on allele *e245*, which causes a G347R mutation in UNC-17. Two of the suppressors (*sup-1* and *sup-8/snb-1*) have previously been shown to encode synaptic proteins able to interact directly with UNC-17. We found that the remaining suppressor, *sup-2*, corresponds to a mutation in *erd-2.1*, which encodes an endoplasmic reticulum retention protein; *sup-2* causes a V186E missense mutation in transmembrane helix 7 of ERD-2.1. The same missense change introduced into the redundant paralogous gene *erd-2.2* also suppressed *unc-17(e245)*. Suppression presumably occurred by compensatory charge interactions between transmembrane helices of UNC-17 and ERD-2.1 or ERD-2.2, as previously proposed in work on suppression by SUP-1(G84E) or SUP-8(I97D)/synaptobrevin. *erd-2.1(V186E)* homozygotes were fully viable, but *erd-2.1(V186E); erd-2.2(RNAi)* exhibited synthetic lethality [like *erd-2.1(RNAi); erd-2.2(RNAi)*], indicating that the missense change in ERD-2.1 impairs its normal function in the secretory pathway but may allow it to adopt a novel moonlighting function as an *unc-17* suppressor.

## Introduction

Genetic suppression provides a remarkably effective tool for investigating biological function, allowing the exploration of gene and protein interactions, regulatory pathways, as well as revealing basic processes of gene expression. Suppression analysis has been especially powerful when applied to the model organism *Caenorhabditis elegans*, as a consequence of the small size of this animal, its diploidy, and its reproduction by self-fertilization, features which allow examination of large numbers of individuals and the isolation of both dominant and recessive suppressor mutations (Hodgkin 2005).

**Figure 1:**
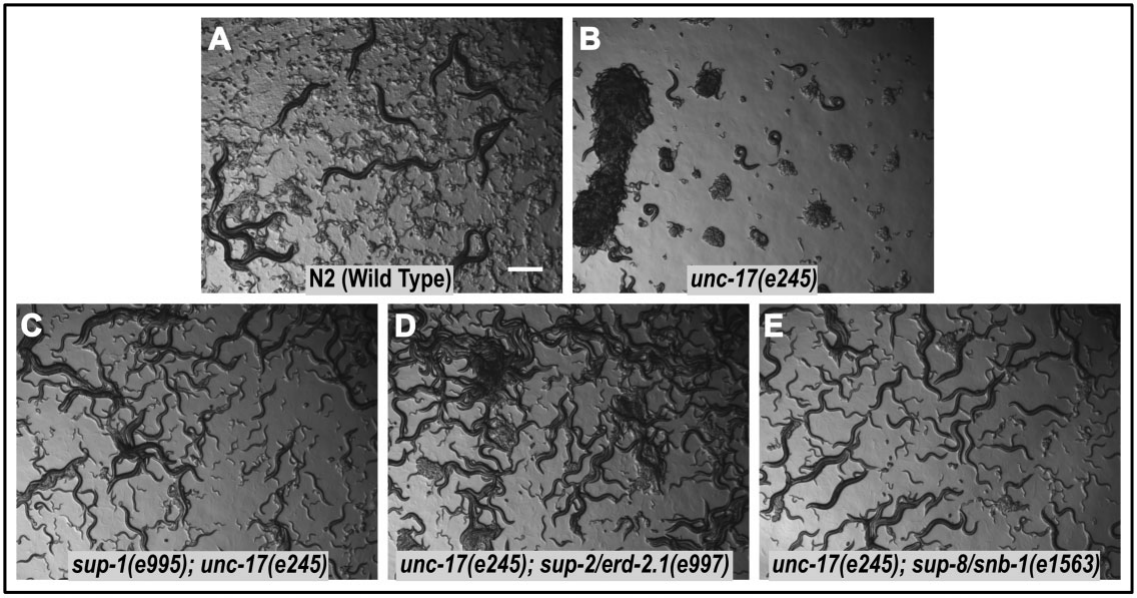
Wild-type, uncoordinated, and suppressed phenotypes. Images of mixed-stage *C. elegans* late exponential populations of the indicated genotypes. Scale bar ∼0.5 mm.

Suppression mechanisms can be informational, gene- or allele-specific. One example of the latter is provided by mutations of the gene *unc-17* and their suppressors, which have been productively studied for many years (Sandoval *et al.* 2006; Mathews *et al.* 2012). The *C. elegans* gene *unc-17* encodes an acetylcholine transporter, required for loading this transmitter into synaptic vesicles and consequently essential for all cholinergic neurotransmission in this animal (Alfonso *et al.* 1993). Null mutants of this gene are lethal, but many viable hypomorphic mutants have been isolated, most of which were resistant to acetylcholinesterase inhibitors such as lannate or aldicarb (Brenner 1974; Miller *et al.* 1996). Most also exhibited severe locomotory defects; among these was the mutant *e245*, which expresses a strong coiler phenotype and very limited mobility at all developmental stages. In pilot experiments by Sydney Brenner, it was found that further mutagenesis of *e245* strains could yield rare animals with apparently normal movement, indistinguishable from wild type (Figure 1). These animals were detected in the first generation after mutagenesis, and segregated self-progeny in a wild-type: Unc ratio of 3:1, indicating dominance. The extreme difference in mobility between the Unc animals and revertants meant that the latter could be detected at frequencies as low as 10^−5^. For example, one normally moving animal could readily be found and picked from a 9 cm plate containing at least 10^4^ uncoordinated worms (Hodgkin 1974).

Different mutagens were tested for efficacy: it was found that both EMS (ethyl methanesulfonate) and (much less efficiently) UV or X-irradiation could yield strong, apparently wild-type revertants (Hodgkin 1974). No spontaneous strong revertants were observed, despite continuous culture of *unc-17(e245)* strains over many years.

The ease with which EMS revertants could be detected prompted the first experiment described below, which addressed the mechanism of EMS mutagenesis in *C. elegans*. The main effect of this agent on DNA is to cause the alkylation of guanine residues, creating O-6-ethylguanine (Sega 1984). This modified base can potentially pair with thymine residues during DNA replication, thereby creating a GC-to-AT transition mutation. However, such mispairing does not necessarily occur immediately, so there may be a delay of several divisions before a mutation is established, or the modified base may be corrected without causing mutation. Delayed fixation of EMS mutations was demonstrated in a classic experiment on bacteriophage T4 (Green and Krieg 1961). The *unc-17(e245)* phenotype reversion system provided an opportunity to test for the same effect in a multicellular organism.

These experiments yielded some of the first extragenic suppressors to be discovered in *C. elegans* genetics: three loci were defined and named *sup-1*, *sup-2*, and *sup-8*. All three suppressors defined in these experiments behaved genetically as dominant allele-specific modifiers, acting only on the *e245* mutation of *unc-17* (Figure 1). Subsequent work on cloning and sequencing of *unc-17* and its many mutants revealed that *e245* causes a G347R change, producing a positive charge in one of the predicted transmembrane helices of the transporter protein, which presumably results in an almost complete lack of activity (Alfonso *et al.* 1993).

Molecular explanations for the extremely efficient suppression of *unc-17(e245)* by its extragenic modifiers emerged from detailed analyses of *sup-8* and *sup-1*, which were found to encode synaptic proteins (Sandoval *et al.* 2006; Mathews *et al.* 2012). *sup-8(e1563)* is a missense mutation affecting SNB-1 (synaptobrevin), a small integral synaptic vesicle membrane protein that is essential for synaptic exocytosis. The *sup-8(e1563)* mutation results in the introduction of an acidic residue (I97D) into the middle of the single transmembrane domain of synaptobrevin. Therefore, restoration of acetylcholine transporter activity could be explained by compensatory charge interactions between the positively charged UNC-17(Arg347) and the negatively charged SNB-1(Asp97) (Sandoval *et al.* 2006).

Similarly, *sup-1* was found to encode a small (103aa) single-pass synaptic protein of uncertain function (Mathews *et al.* 2012). Multiple independent suppressor alleles were found to be identical, all causing the same G84E alteration in the predicted transmembrane domain of SUP-1. Suppression was inferred to occur in a similar manner to *sup-8/snb-1*, by intramembrane interaction between UNC-17(Arg347) and SUP-1(Glu84).

The molecular identity of *sup-2* remained unknown, and is addressed in the present report.

## Materials and methods

### Nematode culture

#### Growth

Standard methods for culture and growth of *C. elegans* were used (Brenner 1974; Stiernagle 2006). Experiments were carried out at 20˚ unless otherwise noted.

#### Strains and nomenclature

Strains utilized were: N2 wild type, BA17 *fem-1(hc17) IV*, CB933 *unc-17(e245) IV*, CB2210 *unc-17(e245) IV; sup-2(e997) X*, CB3031 *unc-17(e245) IV; snb-1(e1563) V*, CB3987 *pha-1(e2123) III*, CB4985 *sup-1(e995) III; unc-17(e245) IV*, CB7427 *unc-17(e245) IV; eEx849[erd-2.2(V186E) + sur-5p::GFP]*, CB7430 *unc-17(e245) IV; eEx855[erd-2.1(V186E) + sur-5p::GFP]*, CB7550 *erd-2.1(e997) X*, CB7551 *rrf-3(pk1426) II; erd-2.1(e997) X*, NL2099 *rrf-3(pk1426) II*, RM3727 *pha-1(e2123) III; unc-17(e245) IV; mdEx1160[unc-17p::erd-2.1(V186E) + pBX].*

Unless otherwise specified, the term *“sup-8/snb-1”* refers to the allele *e1563*, and *“sup-2/erd-2.1”* refers to the allele *e997*. Relevant new strains generated in this study are available from the Caenorhabditis Genetics Center.

#### Synchronization

Pure populations of homozygous *unc-17(e245)* L1 larvae were obtained in two ways. For the first experiment, plates of starved *unc-17* worms that had accumulated large numbers of arrested L1 larvae were washed off with M9 buffer and the resulting suspensions were taken through repeated cycles of settling under normal gravity in 5 ml volumes of M9, in order to remove all developmental stages later than the slow-to-settle L1 animals. For subsequent experiments, adult populations were bleached and sheared to produce pure egg populations, which were then hatched in M9 buffer overnight to generate synchronous arrested L1 larvae (Stiernagle 2006).

#### Mutagenesis

For EMS mutagenesis, worms were washed with M9 buffer and incubated for 4 h in 0.05 M EMS dissolved in M9 buffer (Brenner 1974). For UV mutagenesis, washed worms were spread on 9 cm NGM plates and irradiated for 60–300 s using a calibrated UV source delivering 1 W/s/m^2^. For X-ray treatment, washed worms were spread on 9 cm NGM plates and exposed for 5–10 min to an X-ray source delivering 500 rad/min.

#### Movement assays

**Figure 2:**
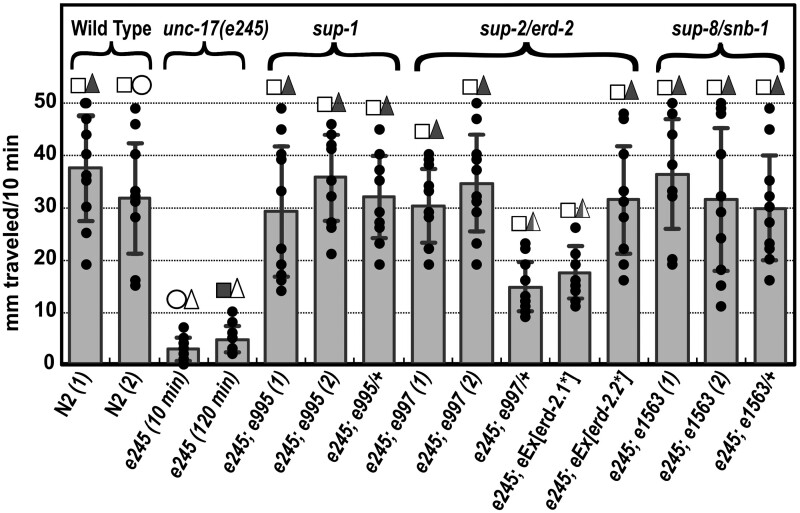
Relative mobility: crawling on solid media. Procedural details are in the Materials and Methods section. *e245* is an *unc-17* allele, *e995* is a *sup-1* allele, *e997* is a *sup-2/erd-2.1* allele, and *e1563* is a *sup-8/snb-1* allele. Suppressing transgenes are abbreviated as eEx[erd-2.1*] and eEx[erd-2.2*]; the asterisks indicate that the encoded proteins carry the V186E amino acid substitution. Filled black circles represent the distances traveled by each animal, and error bars indicate the standard deviations for each set of 10 measured values. Confidence limits for the data sets are indicated by the squares and triangles over each data set, as follows. Squares designate *P*-values comparing a given data set with the 10-min *e245* data set (open circle)—filled squares indicate *P* > 0.1, unfilled squares indicate *P* < 0.0005. Triangles designate *P*-values comparing a given data set with the N2 data set #2 (open circle)—filled triangles indicate *P* > 0.2, partially filled triangles indicate *P* < 0.005, unfilled triangles indicate *P* < 0.0005. Statistical significance was determined using the two-sample Mann-Whitney *U*-test.

Crawling mobility on solid media (Figure 2) was assayed by placing 15 adult hermaphrodites on one end of a 50 × 5 mm lawn of *Escherichia coli* spread on NGM agar, and measuring the distances moved by the 10 “fastest” animals after 10 min. Statistical significance was determined using the two-sample Mann-Whitney *U*-test.

### Immunofluorescence staining

Nematodes were fixed and stained as previously described (Mullen *et al.* 2006), using anti-UNC-17 antibodies: mouse monoclonal mAb1403, chicken polyclonal C96, or rabbit polyclonal R383. Some batches were double-stained with mouse monoclonal antibody 1401 or 1415 to ChAT (choline acetyltransferase) (Duerr *et al.* 2008) as an internal control for the staining intensity (these antibodies also label cholinergic cells). Comparisons of anti-UNC-17 staining intensity were done blind by eye on 3–5 batches of strains fixed, stained, and imaged together. To confirm these observations, matched 512 × 512 (100 × 100 microns) confocal images of the nerve ring were taken blind to the genotype. Collection parameters were set so that the individual sections did not have any saturated pixels (*i.e.*, gray values < 255). A maximum projection tiff file was created and the 25-pixel circle within the nerve ring with the highest average intensity was identified. For each experiment, the highest average intensities in this circle for each strain were averaged (1–6 worms per genotype). These data supported the observed order of immunoreactivity: *unc-17(e245) < unc-17(e245); sup < sup* for each of *sup-1(e995), sup-2/erd-2.1(e997),* and* sup-8/snb-1(e1563).*

### Transgenesis

Procedures for microinjection and selection or identification of transformants were as described (Praitis and Maduro 2011). A wild-type genomic clone of *erd-2.1* was modified by site-directed mutagenesis to encode ERD-2.1(V186E) and fused to the 5 kb *unc-17* promoter; this promoter is expressed specifically in (and defines) cholinergic neurons (Serrano-Saiz *et al.* 2020). The resulting construct was injected into *pha-1(e2123*ts*)* animals together with pBX, a *pha-1(+)* clone (Granato *et al.* 1994), allowing selection of transformants at restrictive temperature. Transformed animals were crossed with *unc-17(e245)* and a suppressed line, RM3727 = *pha-1(e2123*ts*); unc-17(e245); mdEx1160[unc-17p::erd-2.1(V186E) + pBX]* established from subsequent progeny. Longer genomic clones of *erd-2.1* and *erd-2.2* that included presumed endogenous promoter regions were similarly modified to encode ERD-2.1(V186E) and ERD-2.2(V186E), and injected into wild-type animals (at 3 ng/µl) using *sur-5p::GFP* (pTG96) as a transformation marker (at 90 ng/µl). For *erd-2.1*, 6.6 kb including 4.7 kb of upstream intergenic sequence was cloned from wild-type DNA using primers aggaaacgcgtaaacgagtaacgc (erd-2.1For) and cccgcaggaaacaacaatcgatcc (erd-2.1Rev), and modified using primer gctggaatcGAGcaaaccgtt (erd-2.1V186EFor). For *erd-2.2*, 5.0 kb including 3 kb of upstream intergenic sequence was cloned from wild-type DNA using primers acgatcacagtcgtcacagaagagc (erd-2.2For) and cgtcgtttctccgagactttccaag (erd-2.2Rev), and modified using primer gccggaattGAAcaaactgtt (erd-2.2V186EFor). New England Biolabs Site-Directed Mutagenesis Kit E0554 was used for modification. The resulting extrachromosomal transgenes (*eEx849 [erd-2.2(V186E) + sur-5p::GFP], eEx855 [erd-2.1(V186E) + sur-5p::GFP])* were then crossed into *unc-17(e245)* to establish suppressed lines. Three or four independent transgenes were tested for each construct. Strains CB7427 = *unc-17(e245) IV; eEx849[erd-2.2(V186E) + sur-5p::GFP]* and CB7430 = *unc-17(e245) IV; eEx855[erd-2.1(V186E) + sur-5p::GFP]* were used for the measurements shown in Figure 2.

### RNAi knockdown

All RNAi experiments were performed at 20°C by the feeding method (Kamath *et al.* 2003) using *C. elegans* strains N2 (wild-type), CB7550 *[sup-2(e997)]*, NL2099 *[rrf-3(pk1426)]*, and CB7551 *[rrf-3(pk1426); sup-2(e997)].* HT115 *E. coli* clones containing L4440 plasmids coding for the dsRNAs of interest (*erd-2.1* and *-2.2*) were selected from the Ahringer RNAi library (Kamath *et al.* 2003). Sanger sequencing using M13 primers (performed by Source Bioscience) confirmed the identity of these clones. Bacteria from frozen glycerol stocks were used to streak 2XTY plates containing ampicillin (100 μg/ml) and tetracycline (15 μg/ml), which were then incubated overnight at 37°C. Single colonies from those plates were used to inoculate 6 ml of liquid 2XTY medium plus ampicillin. Bacterial cultures were grown at 37˚C and used to seed NGM RNAi agar plates (55 mm, 100 μg/ml ampicillin, 1 mM IPTG). For the synchronous knockdown of both *erd-2.1* and *-2.2*, equal volumes of the respective cultures were mixed together before seeding. Mixtures with unequal ratios (1:9 or 9:1) were found to be as efficacious as 1:1 mixtures. A single OP50-fed young L4 hermaphrodite (P_0_) was transferred onto each seeded plate and then removed after 48 h. Three to five separate broods were examined for each condition. Progeny was examined for survival and abnormal phenotypes. RNAi-knockdown of *pop-1* (conferring an embryonic lethal phenotype) was used as a positive control for the uptake and expression of dsRNA. Brood size, hatching and larval survival beyond L2 stage are shown in Supplementary Table S2.

## Data availability

The data underlying this article are available in the article and in its online Supplemental Material. Supplementary Table S1 contains results of RNAi experiments, and Supplementary Table S2 summarizes growth parameter comparisons. Supplemental Material available at figshare: https://doi.org/10.25386/genetics.14438975.

## Results

### Delayed fixation of mutational events

The *unc-17(e245)* suppression paradigm permitted a test of the phenomenon of delayed fixation of EMS mutations, originally observed in bacteriophage T4 (Green and Krieg 1961). If alkylation of *C. elegans* DNA by EMS were immediately mutagenic, then treatment of L1 larvae should yield large clones of F_1_ progeny carrying a specific mutation, because the germ-cell pool in L1 larvae consists of only two diploid cells (Sulston and Horvitz 1977; Kimble and Hirsh 1979). But if mispairing between the alkylated guanine and a thymine did not always occur at each DNA replication, then fixation might not occur until after several cell divisions, resulting in much smaller clones.

In initial experiments, L1 populations of *unc-17(e245)* animals were collected, exposed to EMS, washed and plated on 9 cm bacterial lawns, at multiplicities of 170–1000 per plate. Plates were incubated until egg-laying began and then, over the next six days, examined carefully for non-Unc F_1_ progeny animals. All non-Unc animals were picked; all exhibited a sustained apparently wild-type locomotory phenotype. Out of 97 plates, seeded with an average of 510 L1 worms per plate, 29 plates produced at least one non-Unc F_1_ animal. All such plates were re-screened repeatedly, to detect all members of a mutant clone.

Results are summarized in Table 1, which shows that most clones (20/29) were small (1–5 animals). Exposure of L1 *e245* worms to the standard dose of EMS resulted in a reduction in self-fertility, from 190 (control) to 120 F1 worms per animal (means, *n* = 8), so the largest clone of revertants (27 animals) may have been as much as 25% of a single brood. However, it is likely that this apparent large clone represented two independent broods occurring on the same plate. Most of the clones detected were much smaller, indicating that fixation of a mutational event occurred at some variable number of divisions after the initial exposure to mutagen.

**Table 1:** F_1_ revertant clone size distribution after L1 mutagenesis

Clone size	Number of occurrences	Number F_1_ heterozygous	Number F_1_ homozygous	Unknown or sterile
0	68	—	—	—
1	3	3	0	0
2	6	8	1	3
3	5	12	0	3
4	3	4	0	8
5	3	15	0	0
6	4	15	1	8
8	2	9	3	4
9	1	0	0	9
11	1	9	0	2
27	1	0	0	27
Total	97	75	5	64

Eighty of the 144 F_1_ revertant worms were picked to separate plates and allowed to produce F_2_ self-progeny. Most segregated both Unc and non-Unc worms, in approximately 1:3 ratio, indicating that the parent worm was heterozygous for a dominant suppressor mutation. A minority segregated only non-Unc worms, indicating homozygosity in the parent, and therefore that the induced mutation had contributed to both oogenic and spermatogenic pools in the hermaphrodite germ-line. This observation showed that homozygous mutants could be generated in the F_1_ generation after mutagenesis, but only at low frequency.

When more mature *e245* animals were mutagenized, some of their rare non-Unc revertant progeny failed to breed true and produced F_2_ broods consisting only of Unc worms. Most probably such worms were genetically mosaic, with germlines in which the suppressor mutation had been lost or corrected while the soma remained mutant. A further experiment showed that such mosaicism is frequent when the parent worms were mutagenized at a late larval or adult stage. *unc-17(e245)* worms were synchronized at L1 stage and equal numbers were allowed to develop for 0–4 days at 15°C before mutagenesis, corresponding to mutagenesis at each of the four larval stages and young adulthood. F_1_ broods were then screened for non-Unc animals and all were picked, as in the first experiment. As shown in Table 2, treatment of young adults generated mostly mosaic revertants, indicating that mutagenesis at this stage is impractical for mutant screens or selections.

**Table 2:** Late mutagenesis produces mosaic progeny

Stage mutagenized	Non-Unc F_1_ progeny	Heterozygous F_1_ progeny	Homozygous F_1_ progeny	Sterile F_1_ progeny	All Unc F_2_ progeny
L1 larva	18	13	1	4	0
L2 larva	32	27	2	3	0
L3 larva	15	11	2	1	1
L4 larva	13	7	1	2	3
Young adult	13	1	0	1	11

Mutagenesis even at L3 or L4 stage yielded some homozygous revertant progeny, confirming the previous inference that spermatogenic and oogenic germlines do not become segregated early in larval development.

Attempts to repeat these experiments using mutagens expected to have more immediate effect, such as UV or X-irradiation, were not successful, owing to much lower efficiency of mutagenesis (Hodgkin 1974). However, rare strong suppressors of *unc-17(e245)* were recovered using either of these agents.

### Characterization of suppressor mutations: sup-1, sup-2, and sup-8

All of the strong suppressors appeared to be extragenic and conferred almost complete restoration of locomotion and motility to *unc-17(e245)* homozygotes (Figures 1 and 2). Some revertant populations generated in later experiments exhibited improved but still abnormal movement; these were found to carry second-site missense mutations in *unc-17*.

Many of the EMS-induced extragenic suppressors generated in the above experiments were mapped genetically (Hodgkin 1974). Most (17/18) exhibited strong linkage (approximately 3% recombination) to the marker *dpy-18* on the right arm of LGIII (Linkage Group III). The complete dominance exhibited by these suppressors precluded complementation testing, so it was assumed that all defined the same locus, designated *sup-1*. One suppressor exhibited strong linkage to the sex-linked marker *dpy-6*, defining a second suppressor locus, *sup-2*. One X-ray-induced suppressor also exhibited sex-linkage but was not further analyzed.

A strong UV-induced suppressor was found to map to a separate location, tightly linked to *dpy-11* on LGV. This defined a third suppressor, *sup-8*.

All three suppressors exhibited allele specificity, acting to restore almost normal movement to *unc-17(e245)* but having no effect on other tested alleles of this gene (Sandoval *et al.* 2006; Mathews *et al.* 2012). For *sup-1* and *sup-8*, tested alleles included missense alleles such as *e283, e335, e464* and *e795* and promoter mutants such as *e876*. For *sup-2*, only *e876* and the identical allele *e113* were tested, as well as *cha-1* alleles (*b401* and *p1152*). Independent isolates of the *unc-17(G347R*) mutation *(e245*, *e359*, and *p300*) were all well suppressed by each of the three suppressors. Limited tests using mutants in a variety of other genes showed no suppressive effects, indicating that these were gene-specific suppressors, in contrast to *sup-5* and other informational suppressors that have been studied in *C. elegans* (Waterston and Brenner 1978; Hodgkin *et al.* 1989).

### Identification of sup-2 as an erd-2.1 allele

Initial mapping by 2- and 3-factor crosses placed *sup-2(e997)* at +1.36 ± 0.36 cM on LGX, corresponding to an interval of 9.30–9.85 Mb on the complete X-chromosome genomic sequence (The C. elegans Sequencing Consortium 1998). Whole-genome sequencing of strain CB2110 [*unc-17(e245); sup-2(e997)]* revealed a total of 112 base changes on the X chromosome, three of which lay in this interval and three just outside it. Of these six candidates, a Val-to-Glu missense change in F09B9.3, now named *erd-2.1* (genetic location +1.86 cM, sequence location 10,147,500) seemed most plausible. The relevant nucleotide change in *erd-2.1* was a T to A transversion (GTG Val → GAG Glu), unlike the G to A transitions most commonly induced by EMS (Sega 1984; Flibotte *et al.* 2010), which was consistent with the rarity of EMS-induced *sup-2* mutations.

A transgene encoding this mutant form of ERD-2.1 (derived from CB2110 genomic DNA) and driven by the *unc-17* promoter was constructed and injected into non-Unc animals, then crossed into *unc-17(e245)* animals. Homozygous *unc-17(e245)* animals carrying this transgene were non-Unc, indicating that suppression was occurring.

Suppression by *erd-2.1(V186E)* was confirmed by using a different transgene carrying 6.6 kb of wild-type genomic sequence encompassing *erd-2.1* plus 4.7 kb of upstream sequence, with the appropriate single base change introduced by site-directed mutagenesis, and *sur-5p::GFP* as a transformation marker. When this transgene was crossed into a homozygous *unc-17(e245)* background, all fluorescent animals were non-Unc and all nonfluorescent animals were Unc, confirming suppression. Three out of three independent transformed lines exhibited suppression. Fluorescent animals from one of these lines were picked for the assays shown in Figure 2, which revealed consistent suppression [albeit weaker and more variable than with *unc-17(e245); sup-2(e997)* = CB2110].

### Suppression by erd-2.1 and erd-2.2

The *C. elegans* genome contains two genes with homology to *Saccharomyces cerevisiae ERD2*: F09B9.3 and C28H8.4 (ERD2 has 44% amino acid sequence identity to both). These genes have therefore been given the names *erd-2.1* and *erd-2.2*. The encoded proteins both contain 213 amino acids and are 68% identical, 84% similar in amino acid sequence. Previous work Tischler *et al.* (2006) showed that RNAi knockdown of either of these two genes had no obvious phenotypic consequences, but that simultaneous knockdown of both was lethal, demonstrating that the two genes act redundantly in an essential process. This essential process is presumably the retrieval of ER proteins that have been trafficked to the Golgi apparatus, as demonstrated for *S. cerevisiae ERD2* (Semenza *et al.* 1990) and Drosophila *KdelR* (Abrams *et al.* 2013).

In view of this redundancy and the sequence similarity of the two proteins, it seemed possible that the same Val-to-Glu change present in *e997* mutants, if introduced into *erd-2.2*, would also suppress *unc-17(e245)*. To test this possibility, an *erd-2.2(V186E)* transgene was constructed from a 5 kb genomic clone including *erd-2.2* plus 3 kb of upstream sequence, modified with a single base change introduced by site-directed mutagenesis. This was then injected into worms together with *sur-5::GFP* as a transformation marker, and crossed into an *unc-17(e245)* background. Four out of four independent transformed lines exhibited suppression, demonstrating that *erd-2.2* can also act as an *e245* suppressor, with similar or possibly greater efficiency to *erd-2.1* (Figure 2).

Suppression by *sup-2(e997)* was detectably weaker than suppression by either *sup-1(e995)* or *snb-1(e1563)*, because heterozygous *unc-17(e245)*; *sup-2*/+ animals moved significantly less well than *unc-17(e245); sup-2* homozygotes (Figures 1 and 2), whereas both *sup-1* and *sup-8/snb-1* suppressors were fully dominant. Suppression by transgenically expressed *erd-2.1(V186E)* and *erd-2.2(V186E)* was also weaker than with *sup-1* and *sup-8/snb-1* (Figure 2), though this may have been partly the result of low levels of expression from the transgenes.

### Mechanism of suppression

**Figure 3:**
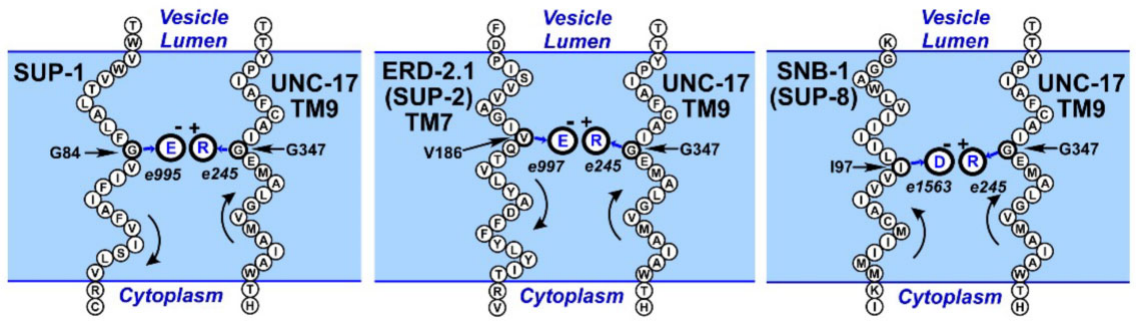
Models for suppressive interaction. Diagrams showing presumed compensatory interactions between transmembrane helices of UNC-17 and suppressor proteins. TM7 sequences are almost identical in ERD-2.1 (PISVVAGIVQTVLYADFFYLYIT) and ERD-2.2 (PIVVVAGIVQTVLYADFFYLYVT). The curved arrows in each panel represent the direction of translation of each protein; note that the SUP-8/SNB-1 transmembrane helix is oriented parallel to UNC-17 TM9, whereas the interacting helices of SUP-1 and SUP-2/ERD-2.1 are antiparallel to UNC-17 TM9.

A crystal structure of the chicken KDELR2 receptor has recently been determined (Bräuer *et al.* 2019). This protein is sufficiently similar to the *C. elegans* ERD-2 proteins to permit confident modeling of the latter (62% amino-acid sequence identity to ERD-2.1, 63% identity to ERD-2.2). The suppressing missense change introduces an acidic residue into the seventh transmembrane helix of the ERD-2 receptor, so it seems likely that the mechanism of suppression is similar to that inferred for *sup-1* and *sup-8/snb-1*. That is, restoration of UNC-17 function or stability is achieved by compensatory charge interactions between the mutant arginine (G347R) in the predicted TM9 of UNC-17, and a mutant acidic residue in a transmembrane domain of SUP-1, SNB-1, ERD-2.1, or ERD-2.2, as shown diagrammatically in Figure 3.

Suppression by mutant ERD-2 has some different features from that seen with SUP-1 and SNB-1, both of which have a single TM domain, whereas ERD-2 has seven TM domains and might therefore have difficulty in interacting closely with UNC-17, which is predicted to have 12 TM domains. However, the altered residue in TM7 of ERD-2 is predicted to lie on the external face of the seven helix bundle (Bräuer *et al.* 2019), so direct interaction with UNC-17 should be possible. A crystal structure for UNC-17 (or any other vesicular acetylcholine transporter) has not yet been determined, but modeling based on related transporters suggests that the mutant arginine residue in TM9 of UNC-17 should also face outwards, permitting interaction with suppressor proteins. Such models are supported by our previous experimental data that the mutant arginine is able to interact with the transmembrane domains of mutant synaptobrevin and SUP-1 proteins (Sandoval *et al.* 2006; Mathews *et al.* 2012).

In contrast to neuronally expressed SUP-1 and SUP-8/SNB-1 (synaptobrevin), which are synaptic proteins normally residing in synaptic vesicle membranes and therefore capable of sustained interaction with UNC-17, ERD-2 proteins would be expected to shuttle between the endoplasmic reticulum and the Golgi apparatus in most or all tissues. Opportunities for interaction between these proteins and UNC-17 might be expected to be only transient, during early trafficking and maturation of UNC-17, and suppression of a synaptic protein by a modified ERD-2 is therefore surprising. Possibly, the proposed interaction increases the stability of the protein or allows correction of a defect in folding or post-translational modification. Alternatively, it is possible that the association of the two proteins allows some aberrant trafficking of mutant ERD-2 so that enough ends up in synaptic vesicles to permit functional suppression of UNC-17(G347R).

UNC-17(G347R) must retain a small amount of transporter activity, because *unc-17(e245)* animals are viable and fertile, albeit slow-growing and extremely uncoordinated. In contrast, *unc-17(null)* animals are inviable and die after hatching as immobile L1 larvae (Alfonso *et al.* 1993). However, it was previously demonstrated that simply increasing the number of mutant UNC-17(G347R) by transgene overexpression does not suppress *unc-17(e245)* (Mathews *et al.* 2012).

**Figure 4:**
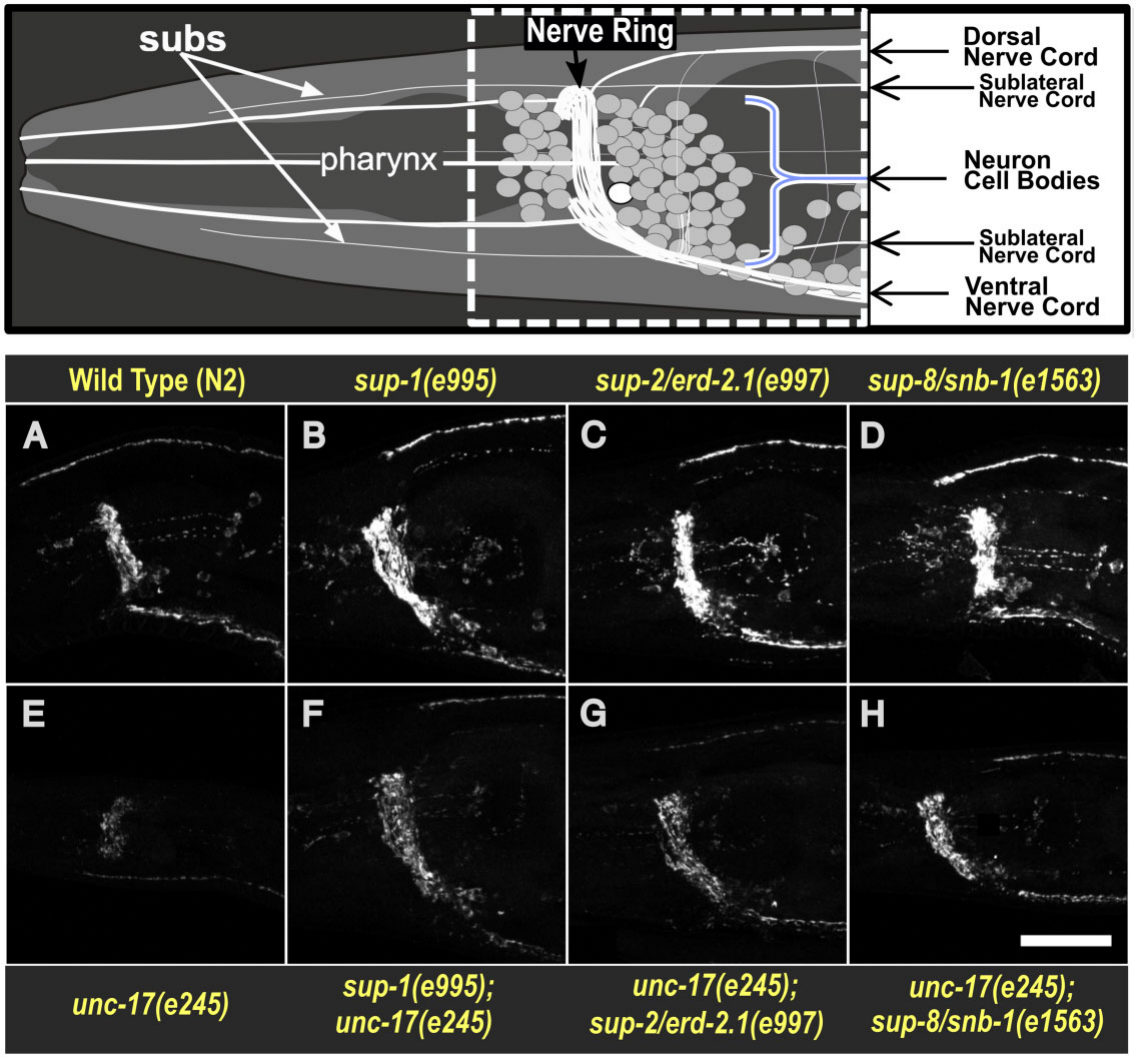
Suppressors of *unc-17(e245)* increase the abundance of UNC-17 protein. Upper panel: diagram of the anterior nervous system of a young *C. elegans* adult, modified from Rand *et al.* (2000). The rectangle with the dashed white line indicates the region of the worm included in the lower images. Panels (A–H) are immunofluorescence images of the nerve ring and ventral and dorsal nerve cords stained with a specific anti-UNC-17 monoclonal antibody (Duerr *et al.* 2008). Because UNC-17 is a synaptic vesicle protein, the immunostaining reflects the locations of cholinergic synapses. Representative examples of animals of each indicated genotype are shown. All nematodes were stained at the same time in the same solutions and imaged on the same day with identical “blind” image collection conditions. Relative intensity in groups of matched strains was evaluated blind 3–5 times by eye and 3 times using confocal microscopy, as described in Materials and Methods section. Anterior is to the left and ventral is down; scale bar is 20 μm.

In order to explore the distribution and levels of UNC-17 in wild-type, mutant and suppressed animals, immunofluorescent staining of UNC-17 was carried out (Figure 4). This showed that little protein could be detected in synaptic regions in *unc-17(e245)* mutants (Figure 4E), but that substantial synaptic staining could be seen in suppressed animals (Figure 4, F–H), similar to wild type.

Surprisingly, UNC-17 staining of *sup-1, sup-2/erd-2.1*, and *sup-8/snb-1* animals in an *unc-17(+)* background appeared possibly more intense than in completely wild-type animals (Figure 4, B–D*vs*Figure 4A), suggesting that there may even be some interaction between suppressor proteins and wild-type UNC-17, which results in higher levels of UNC-17 in synaptic regions.

### ERD-2 redundancy and moonlighting

Analysis of KDEL receptor function based on the crystal structure of the chicken protein revealed that the mechanism of this receptor, and its shuttling between ER and Golgi, depends crucially on properties of the last transmembrane helix, TM7 (Bräuer *et al.* 2019). Sequence alterations in this helix result in a nonfunctional protein, which cannot be retrieved from the Golgi apparatus and instead may get trafficked further along the secretory pathway (Bräuer *et al.* 2019). The sequence alteration in TM7 of the suppressing ERD-2 proteins might therefore be expected to abolish their normal function of retrieving ER proteins from the Golgi. Redundancy between ERD-2.1 and ERD-2.2 would permit viability even if one of these proteins lost its normal function.

**Figure 5:**
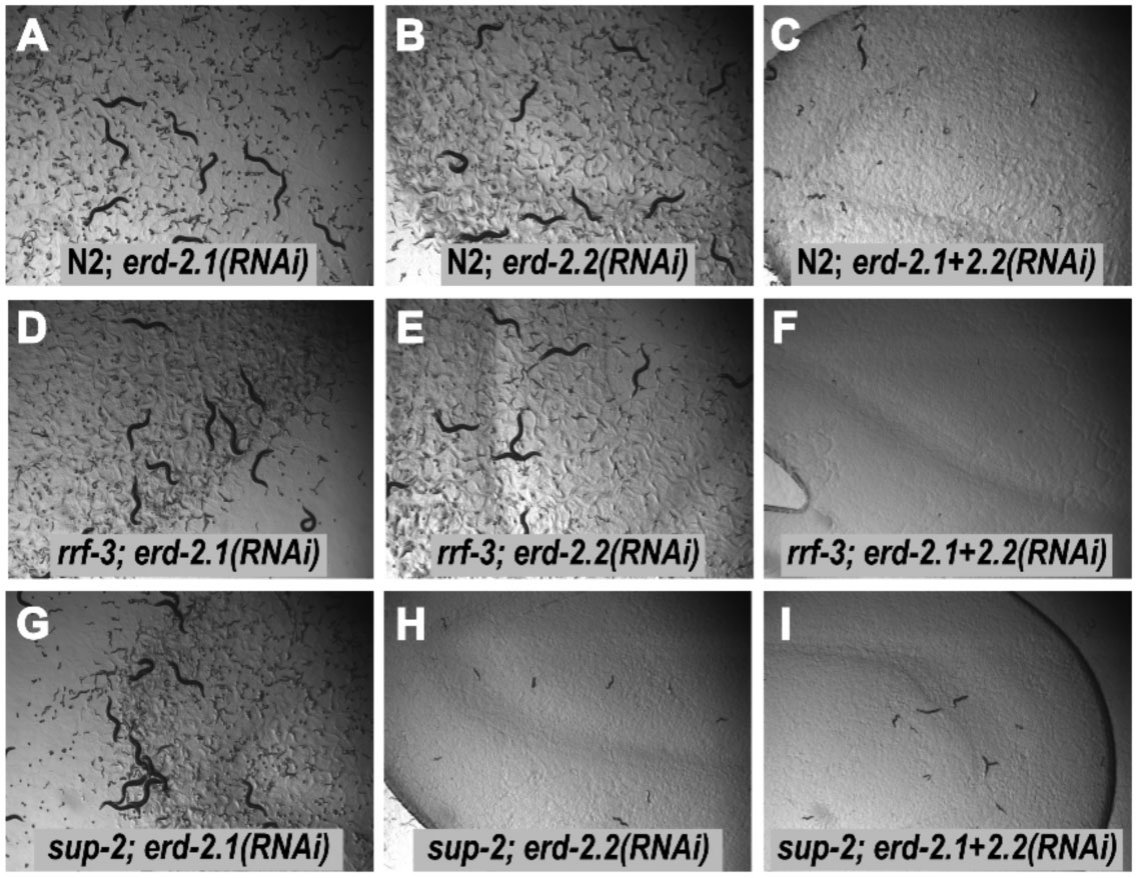
RNAi knockdowns of *erd-2.1* and *erd-2.2* demonstrate synthetic lethality. Images show F_1_ and F_2_ progeny of single animals grown on dsRNA-expressing bacteria, with genotypes as indicated.

We tested this possibility by carrying out RNAi knockdowns of *erd-2.1* and *erd-2.2*, in wild-type and *sup-2* backgrounds (Figure 5). We confirmed the previous observation that knockdown of either gene alone had no obvious effect (Tischler *et al.* 2006), even in an *rrf-3* (enhanced RNAi) background, whereas the double knockdown is lethal, with similar effects in both wild-type and *rrf-3* backgrounds. Many embryos die before hatching, and few animals develop beyond L1 larval stage. In contrast to the viability of *erd-2.2(RNAi)* in a wild-type background, RNAi knockdown of *erd-2.2* in a *sup-2* background resulted in lethality (Figure 5, Supplementary Table S1). Levels of lethality appeared similar in *erd-2.2(RNAi); erd-2.1(RNAi)* and *erd-2.2(RNAi); erd-2.1(V186E).* Repeating these experiments in a *rrf-3* background resulted in a small enhancement of lethality (data not shown). It remains possible that both *erd-2.1(RNAi)* and *erd-2.1(V186E)* retain a low level of ERD-2 receptor activity, insufficient to compensate for the loss of *erd-2.2*.

These observations indicate that ERD-2.1(V186E) has lost most or all of its normal function, which would be consistent with the evidence for the essential importance of residues in helix 7. Nonfunctional KDEL receptors appear to be trafficked to compartments further along the secretory pathway, rather than being retrieved to the ER. It is therefore likely that ERD-2(V186E) gets similarly misdirected, which may allow sustained interaction between the abnormal ERD-2.1(V186E) and UNC-17(G347R), and consequent restoration of transporter function to UNC-17. If so, it is only by losing their normal function that ERD-2 proteins can take on a moonlighting role (Jeffery 2020) as *e245* suppressors.

It is not obvious why ERD-2 function is shared between two redundant genes in *C. elegans* and most other animal species. In the hope of identifying some function uniquely provided by *erd-2.1*, we crossed *erd-2.1(e997)* onto an *unc-17(+)* background to generate strain CB7550, and compared various properties of this strain with the wild-type parental strain N2, but observed no obvious difference in size, mobility, growth rate, hermaphrodite fertility, dauer formation, male mating efficiency or sensitivity to heatshock or starvation (Supplementary Table S2).

We also tried to test the relative importance of the two genes by varying the input ratios of dsRNA-expressing bacteria in the double knockdown experiments, but observed the same levels of lethality when either of the RNAi feeding strains was provided in nine-fold excess.

## Discussion

### Characteristics of EMS mutagenesis

EMS was found to be a remarkably efficient and convenient mutagen for research on *C. elegans* (Brenner 1974), and as a consequence it has been used to induce most of the laboratory mutations generated in research on this model system. Ease of mutagenesis has been one of the most convenient features of *C. elegans* experimentation; moreover, the organism is diploid and reproduces primarily by self-fertilization, allowing the ready detection of both recessive and dominant mutations. In addition, its small size and rapid growth means that for many purposes it can be treated as a microorganism, permitting the handling and screening of large numbers of individuals, and correspondingly allowing the detection of rare genetic events. These properties were exploited in this study.

The initial impetus for the work described in this study was to utilize the convenience of *unc-17(e245)* suppressor detection to explore the effect of different mutagens and to test whether EMS mutagenesis exhibits the same delayed fixation that had previously been shown for mutagenesis of T4 bacteriophage (Green and Krieg 1961). This was found to be the case: even when animals were mutagenized at the first larval stage, when only two diploid germ cells are present, the number of F1 progeny carrying a *sup-1* suppressor mutation is usually small or very small, indicating that the fixation event had only occurred after several mitotic divisions. If fixation occurred at the time of exposure to EMS, when the relevant DNA alkylation is most likely to have taken place, then a quarter of the self-progeny brood (on average 120 animals, after treatment of L1 larvae with EMS) would be expected to express a dominant suppressor mutation and therefore exhibit wild-type locomotion. Only one out of 29 suppression events detected gave rise to a clone of this size, and even this one may have been a double event. In principle, the results listed in Table 1 could be used to infer a probability of fixation at each germline mitotic division, although the numbers are too small to justify detailed analysis.

Other mutagens, for which more immediate fixation might be expected, were tested, but these proved to be too inefficient to be used with this experimental paradigm. However, both UV and X-rays appeared to generate a different spectrum of suppressor events from EMS. Suppression of severe Unc mutants has been used subsequently to explore mutation both by chemical agents and by genetic mutators, most notably by making use of the muscle mutant *unc-93(e1500)* (Greenwald and Horvitz 1980). ENU (N-ethyl-N-nitrosourea) has been found to be an especially useful mutagen for generating a wider spectrum of missense mutations than EMS (De Stasio *et al.* 1997).

Two other features of these initial results deserve comment. First, a few F_1_ revertant animals were found to be homozygous for a suppressor mutation, showing that a mutated mitotic germ cell had contributed to both oogenic and spermatogenic pools in the parent animal. Such homozygotes were detected even after mutagenesis at L3 or L4 stages, suggesting that the two germline pools are not segregated and can continue to mix throughout larval development. Second, mutagenesis of animals at late larval or young adult stages gave rise to many revertant progenies, which displayed normal movement but produced only Unc progeny (Table 2). Delayed fixation explains the existence of these animals: they were presumably genetically mosaic, with the suppressor being fixed only in somatic lineages but not in the germline. The frequency of such events means that mutagenesis at late stages may be inappropriate in some schemes for mutant screening or selection using *C. elegans*. Similar mosaicism after EMS treatment has also been reported in Drosophila (Sega 1984).

### Nature of suppressors

The molecular nature of the allele-specific suppression of *unc-17(e245)* has been previously elucidated for two of the suppressors, *sup-8* (Sandoval *et al.* 2006) and *sup-1* (Mathews *et al.* 2012). In the present report, we identified the third suppressor, originally named *sup-2*, as a missense mutation of *erd-2.1*, affecting protein ERD-2.1, which is expected to act as a retrieval protein for endoplasmic reticulum proteins with C-terminal HDEL or KDEL sequences. A transgene expressing the same missense change in the paralogous gene *erd-2.2* was also found to suppress *unc-17(e245)*, showing that either *erd-2.1(V186E)* or *erd-2.2(V186E)* can suppress.

Suppression by this third class of protein has both similarities and differences from the previously analyzed cases of *sup-8/snb-1* and *sup-1*. In all three situations, suppression depends on the introduction of an acidic residue near the middle of a transmembrane domain, which should allow electrostatic charge interaction with the mutant basic residue in TM9 of UNC-17(G347R). This interaction may act to restore either stability or function or both, to the UNC-17 acetylcholine transporter. In the absence of suppression, levels of mutant UNC-17 are reduced, but it has been established that simple overexpression of UNC-17(G347R) does not correct the mutant phenotype (Mathews *et al.* 2012), so the suppressive interactions must be doing something more than preventing degradation of the mutant transporter.

The compensatory interaction model is plausible, but it is difficult to establish whether direct protein–protein interaction is occurring, and whether such interaction is sustained or only transient. For the case of SUP-1, BiFC (bimolecular fluorescence complementation) tagging of SUP-1 and UNC-17 was used to demonstrate close proximity of these proteins in synaptic vesicles, which is consistent with the model but does not prove sustained direct molecular interaction. (Mathews *et al.* 2012). Comparable BiFC experiments using tagged ERD-2 proteins are conceivable.

### Distinct features of suppression by ERD-2

Suppression by mutant ERD-2 is different from the SNB-1 and SUP-1 cases in several ways. First, the ERD-2 proteins have multiple TM domains, in contrast to the single-pass TM domains of synaptobrevin and SUP-1. This multi-pass structure might hinder the proposed direct interaction between ERD-2 and multi-pass UNC-17. However, the predicted geometries of the two proteins indicate that the charged mutant residues are located on the outside faces of both partners, so intramembrane interaction should be possible. Nevertheless, conditions for the presumed suppressive interactions are not straightforward, because a mutant synaptotagmin with a similar charge change to the suppressing synaptobrevin was found to be an inefficient suppressor (Sandoval *et al.* 2006), despite the expected similar locations and function of synaptotagmin and synaptobrevin.

Second, SNB-1 and SUP-1 are expressed in the nervous system and are known to be located in synaptic vesicles along with UNC-17, permitting sustained or repeated interactions between the suppressor proteins and the transporter, whereas ERD-2 proteins are expected to be mainly or exclusively resident in the ER and Golgi. If the ERD-2 proteins remain in the early compartments of the secretory pathway, then suppression must depend on a transient interaction during the maturation of UNC-17. Alternatively, the mutant ERD-2 proteins may get mislocalized and end up in synaptic vesicles. Establishing the subcellular location of wild-type and mutant ERD-2 proteins in *C. elegans* would require generation of suitable antibodies or creating fluorescently tagged versions of these proteins.

Third, the synthetic lethality of genomic *sup-2/erd-2.1(e997)* and *erd-2.2(RNAi)* indicates that ERD-2.1(V186E) has lost most or all of its normal function, because RNAi knockdown of *erd-2.2* in an *erd-2.1(V186E)* background is lethal, whereas the same knockdown in a wild-type background has no effect. Redundancy between the two *erd-2* genes allows suppression to occur without lethality. In contrast, the suppressing form of SNB-1 is compatible with its normal function as a synaptic exocytosis protein, because *sup-8/snb-1(I97D)* appears to be homozygous viable and normal, whereas *snb-1(null)* is lethal (Nonet *et al.* 1998). For SUP-1, it is not clear whether the suppressing form is functional or not, because the loss of this protein has only subtle effects (Mathews *et al.* 2012). For ERD-2, it is not surprising that the alteration in helix 7 leads to loss of function, because experiments on a vertebrate KDEL receptor have shown that properties of this helix are crucial for its shuttling function (Bräuer *et al.* 2019). Versions of this protein with alterations in TM7 are not retrieved from the Golgi apparatus and instead may get trafficked to more distal compartments along the secretory pathway.

### Moonlighting

This probable misdirection of the mutant protein may in fact explain why ERD-2(V186E) can act as a suppressor at all: it may be that only by losing its normal function that this protein can be trafficked downstream and take on a new function of interacting with UNC-17. If so, the adoption of a novel function for ERD-2 comes with a cost, and is only possible because of the redundancy of *erd-2.1* and *erd-2.2*. Such a change in function can be viewed as an example of protein moonlighting (Jeffery 2020). Alternatively, if the suppressor proteins have entirely lost their normal functions, the causative mutations might be better described as neomorphic changes.

### Redundancy

The presence of two, or sometimes three, paralogs of the KDEL receptor is almost universal in animal genomes, with rare exceptions such as Drosophila (Abrams *et al.* 2013), and remains unexplained. Detailed examination of the properties of an *erd-2.1(V186E)* strain revealed no obvious differences from wild type, so the reason for the redundancy is unclear. Whether this apparent lack of a detectable phenotype is also true of *erd-2.2(V186E)* remains to be tested. Further experimentation on the *C. elegans erd-2* genes is feasible and may allow detailed *in vivo* investigation of ER and Golgi function in this model system, as well as more stringent tests of apparent redundancy between *erd-2.1* and *erd-2.2*. As shown in an extensive analysis (Tischler *et al.* 2006), redundancy between some gene duplicates in Caenorhabditis, such as the *erd-2* genes, appears to have been maintained for many millions of years, despite the expectation of evolutionary loss or functional divergence.

Redundant or partly redundant gene families, both small and large, are a common feature of eukaryotic genomes. One incidental advantage of such redundancy may be that it enables a wider spectrum of mutational possibilities, by permitting survival of otherwise deleterious mutants, as in the present instance.

### Physiological significance

A remaining question is whether the strong suppression effects described in this and previous papers (Sandoval *et al.* 2006; Mathews *et al.* 2012) reveal protein–protein interactions that are physiologically important under normal circumstances. Intramembrane interactions between wild-type proteins may have subtle effects on the function of the interacting partners and therefore be intrinsically advantageous. An alternative model is that suppression is occurring simply as a consequence of the crowded environment of the synaptic vesicle membrane, which may force integral membrane proteins into close proximity and thereby allow fortuitous interactions between neighbors (Mathews *et al.* 2012). The fact that synaptic levels of wild-type UNC-17 appear to be increased by the suppressor proteins, as shown in Figure 4, A–D, suggests that interactions can occur even in the absence of suppressive charge complementarity.

Nevertheless, even though none of these suppression effects may have relevance to the normal life of the organism, they may become important in a long-term context. Rare missense mutations, such as those we have described, may result in novel protein–protein interactions with unusual phenotypic consequences and thereby open up new realms of biology for evolutionary exploration.
